# Life post-COVID-19: symptoms and chronic complications

**DOI:** 10.1590/1516-3180.2021.139104022021

**Published:** 2021-02-24

**Authors:** Paulo Manuel Pêgo Fernandes, Alessandro Wasum Mariani

**Affiliations:** I PhD. Full Professor, Thoracic Surgery Program, Instituto do Coração (InCor), Hospital das Clínicas da Faculdade de Medicina da Universidade de São Paulo (HC FMUSP), São Paulo (SP), Brazil.; II PhD. Attending Surgeon, Thoracic Surgery Program, Instituto do Coração (InCor), Hospital das Clínicas da Faculdade de Medicina da Universidade de São Paulo (HC FMUSP), São Paulo (SP), Brazil.

The pandemic caused by the virus SARS-CoV-2 is certainly the biggest challenge to global health today. From the first appearance of the disease that it causes (COVID-19) to the present day, according to Johns Hopkins University, 71,792,772 cases and 1,606,685 deaths have been recorded around the world.^1^

As the duration of the pandemic extends and the number of patients who have recovered increases, many authors have been asking what chronic alterations COVID-19 might cause in this population. Cases of patients with persistent symptoms like dyspnea, fatigue, coughing, chest pain, myalgia and arthralgia have been reported in the literature, even among patients whose acute phase of the disease was mild. Other symptoms that have been reported include depression, cognitive disorders, headache and palpitations.^2^

The frequency with which these symptoms persist has not yet been well established, but some studies have shown that it may be high among patients who have recovered from COVID-19. In a study published in JAMA, 143 patients were followed up for an average of 60 days after discharge from hospital and only 18 (12.6%) of them reported absence of symptoms relating to COVID-19, while 32% had one or two symptoms and 55% had three or more.^3^ Repercussions impacting on quality of life occurred in 44.1% of the patients. Halpin et al.^4^ followed up a cohort of 100 patients in the United Kingdom after their discharge, including 32 who had been treated in an intensive care unit (ICU). In the ICU group, 72% continued to have some degree of persistent fatigue, around 50 days after their discharge; in the group treated in wards, persistent fatigue was reported in 60.3% of the cases.

The chronic complications that may persist after infection with SARS-CoV-2 mainly affect the respiratory, cardiovascular, renal and neurological systems.

One of the first studies to explore chronic alterations to the respiratory system caused by COVID-19 was published in June 2020. A total of 57 patients were followed up. They underwent a pulmonary function test, a six-minute walking test and chest computed tomography (CT) 30 days after their discharge from hospital. Tomographic alterations were seen in 31 patients (54.3%). Abnormalities in pulmonary function tests were detected in 43 patients (75.4%). In comparison with non-severe cases, the patients presenting severe disease had higher incidence of impairment of diffusing capacity of the lungs for carbon monoxide (DLCO) (75.6% versus 42.5%; P = 0.019). Diminished DLCO, lower respiratory muscle strength and abnormalities on pulmonary imaging were detected in more than half of these COVID-19 patients who were in the initial stage of convalescence.^5^

These data were not completely corroborated by Lerum et al.,^6^ who published a prospective study on 103 COVID-19 patients, including 15 cases that were considered severe and were treated in an ICU. Their aim was to report on their patient’s quality of life, state of dyspnea, pulmonary function and chest CT findings, three months after their discharge from hospital. They found that a quarter of their patients continued to present opacities on chest CT and diminished diffusion capacity. However, in their sample, this was not reflected in increased dyspnea or impaired pulmonary function. ICU admission was the criterion most associated with the presence of pathological CT findings.

Cardiac alterations have also been targeted in studies. In a cohort study on 100 patients who had recovered from COVID-19, cardiac magnetic resonance imaging (MRI) was performed on average 71 days after the disease had been diagnosed. Cardiac alterations were found in 78 patients and active myocardial inflammation in 60 patients. This occurred independent of the patient’s preexisting conditions, disease severity, general evolution of the acute disease or length of time since the original diagnosis.^7^ Nonetheless, the long-term evolution of such cases remains uncertain.

Rajpal et al.^8^ also used cardiac MRI but studied a very specific population. They recruited 26 university athletes who had had COVID-19. None of them needed hospitalization. Twelve of them (26.9%) reported having had mild symptoms, while the others had been asymptomatic. None of them were found to present any ST/T wave alterations in electrocardiograms, and all of them had ventricular volumes and functions that were within the normal range, through transthoracic echocardiograms and cardiac MRI. None of the athletes presented elevated serum levels of troponin I. Four of them (15%) had cardiac MRI findings consistent with myocarditis. Thus, the study by Rajpal et al. showed that even among asymptomatic individuals who are physically fit, cardiac alterations may occur. However, the importance of these findings remains unknown.

The neurological alteration that has been most reported after COVID-19 is persistence of olfactory dysfunction. Otte et al.^9^ analyzed the sense of smell of 50 consecutive patients, at least three weeks after they had recovered from an acute condition. Among these patients, 94% reported that they had suddenly lost their sense of smell during the course of the disease. At the time of undergoing an olfactory test after their recovery, 38% of the patients still presented a deficiency, while 61.7% of them had completely recovered their sense of smell.

Other neurological alterations that have also been described still need to be studied further to better characterize them. These include alterations of cognition and memory and deregulation of sleep. Some psychiatric alterations have also been reported, such as mood changes involving depression or anxiety.^2^

Other consequences, albeit hypothetical, may also impact the post-COVID-19 population. A study published in the journal *Future Oncology* theorized a potential carcinogenic effect from infection with SARS-CoV-2, especially in the pulmonary tissue, which would possibly translate in the future into increased risk of cancer among these patients.^10^

What can be expected from these chronic alterations, and even how to treat them, remains to be determined. A group of researchers in Recife, Brazil, published an interesting article in which they discussed the potential use of nuclear medicine as a means of mapping the chronic alterations to the lungs, kidneys, heart and endothelium that are caused by SARS-CoV-2.^11^

The pandemic is not over yet. The real damage that it will leave behind will certainly be much greater than what was initially thought. But right now, we need to get ready to treat these patients who have survived COVID-19 and often come back needing treatment for chronic complaints.
